# Unraveling learning characteristics of transformer models for molecular design

**DOI:** 10.1016/j.patter.2025.101392

**Published:** 2025-10-14

**Authors:** Jannik P. Roth, Jürgen Bajorath

**Affiliations:** 1 Department of Life Science Informatics and Data Science, B-IT, LIMES Program Unit Chemical Biology and Medicinal Chemistry, Rheinische Friedrich-Wilhelms-Universität, Friedrich-Hirzebruch-Allee 5/6, 53115 Bonn, Germany; 2 Lamarr Institute for Machine Learning and Artificial Intelligence, Rheinische Friedrich-Wilhelms-Universität Bonn, Friedrich-Hirzebruch-Allee 5/6, 53115 Bonn, Germany

**Keywords:** machine learning, transformer networks, language models, sequence-based drug design, model system for control calculations, transformer learning characteristics

## Abstract

In drug design, transformer networks adopted from natural language processing are applied in a variety of ways. We have used sequence-based generative compound design as a model system to explore the learning characteristics of transformers and determine if these models learned information relevant for protein-ligand interactions. The analysis reveals that sequence-based predictions of active compounds using transformer models required a proportion of at least ∼60% of the original test sequences. Moreover, predictions depended on sequence and compound similarity of training and test data and on compound memorization effects. The predictions were purely statistically driven by associating sequence patterns with molecular structures, thus rationalizing their strict dependence on detectable similarities. Moreover, the transformer models did not learn target sequence information relevant for ligand binding. While the results do not call sequence-based compound design approaches generally into question, they caution against over-interpretation of transformer models used for such applications.

## Highlights

• Transformers can generate active compounds based on target protein sequences
• Sequence similarity between training and test sets is required
• The models memorize training compounds regardless of their activity
• They rely on statistics and do not learn biologically relevant information

## The bigger picture

Transformer networks are the basis for large language models and are also widely used in different scientific fields, including drug design. Given their flexible architecture and signature (self-)attention mechanism, transformers are suitable for many generative modeling tasks. Predictions of transformer models, however, are difficult to rationalize. Indeed, concerns have been raised that these models can sometimes act as “Clever Hans” predictors, evoking a comparison to a famous 19^th^-century horse that appeared to be able to count but was simply reading subtle body language cues from his trainer. Such models may provide desirable results but for reasons different than those anticipated, leading to potentially false understandings of the causal relationships in the system. Here, we have used sequence-based generative compound design as a test system to study the learning characteristics of transformer models. Our findings show that predictions of protein-sequence-based transformer models are purely statistically driven and that care should be taken not to over-interpret these predictions.

## Graphical abstract

**Figure:**
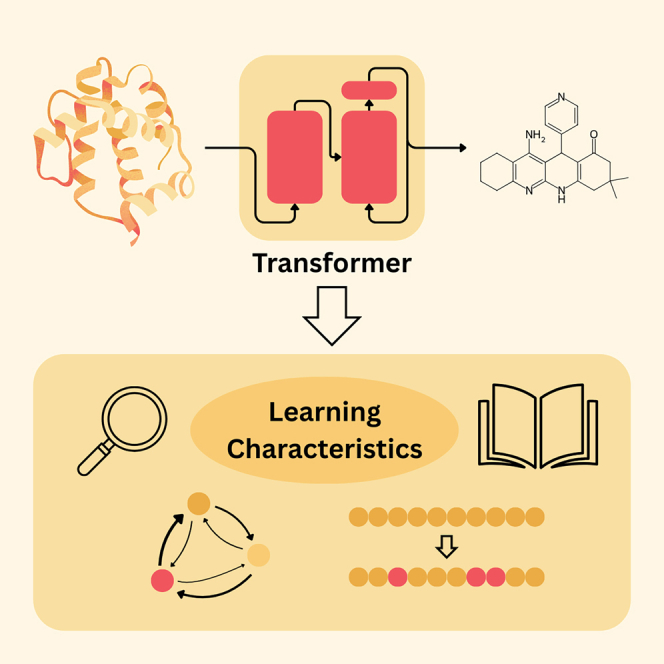

## Teaser

Transformer models are adaptable to off-the-beaten-path molecular design tasks, such as protein-sequence-based compound design. The authors use these predictions as a model system to unravel the learning characteristics of transformers. The analysis reveals that the transformer predictions are statistically driven and that the models do not learn protein-ligand interactions or other biologically relevant information. Instead, compound memorization plays an important role. These findings caution against over-interpretation of sequence-based generative compound design using transformer models.

## Introduction

Deep generative models from natural language processing^1^ have been adopted in numerous scientific fields, including chemistry and drug discovery,^2^ where they are often termed chemical language models (CLMs).^2^^,^^3^^,^^4^ These models operate on textual representations of chemical structures, such as simplified molecular input line entry system (SMILES) strings or other tokenized sequential data.^5^ For CLMs, transformer networks have become a preferred architecture, as in other areas,^6^^,^^7^ mostly due to their hallmark attention and self-attention mechanism,^7^^,^^8^ which has improved predictive performance compared to other neural networks in many applications. Transformer CLMs learn mappings of textual representations of molecules for machine translation tasks, analogous to their original use in natural language processing.^7^ They are applied, for instance, for the generative design of new compounds or the prediction of molecular properties.^9^^,^^10^ However, transformer predictions are difficult to explain and interpret, which often poses a problem for the acceptance of generative modeling in interdisciplinary research. Currently, explainable artificial intelligence (XAI) approaches^11^ for transformers are essentially confined to visualizing attention weights^8^ and analyzing attention flow^12^ or weight gradients.^13^

Notably, the versatility of transformer architectures^6^^,^^7^ in learning different mappings of textual representations of input-to-output molecules is a particularly attractive feature. Furthermore, compound generation can be conditioned by context-dependant chemical rules. Transformers are applicable to off-the-beaten-path molecular design tasks that are difficult, if not impossible, to address using conventional machine learning or other drug design methods.^4^^,^^6^ Protein-sequence-based compound design provides a good example. About two decades ago, the first studies combining sequence and compound information for machine learning were reported.^14^^,^^15^^,^^16^ These early attempts mostly aimed at distinguishing between true and false protein-ligand interactions using neural networks or support vector machine classification models.^14^^,^^15^^,^^16^ However, efforts to directly predict active compounds from sequence data were only rarely reported.^17^ Compared to 3D-structure-based drug design, the scientific rationale underlying sequence-based drug design is not very strong. For instance, only a limited number of residues in proteins participate in ligand binding, similar folds/structures might occur in the presence or absence of detectable sequence similarity, sequence motifs directly associated with the binding of small molecular ligands are not always known and limited in size, and only high global sequence similarity is indicative of similar ligand binding characteristics. Accordingly, designing compounds based on sequence data via machine learning or other computational approaches is challenging. Therefore, it is not surprising that knowledge-driven structure-based methods have dominated drug design since the 1980s, together with ligand-based approaches with origins dating back to the 1960s.

However, following the introduction of transformers in chemistry, a number of sequence-based compound design studies using transformer CLMs have been reported, with promising results in benchmark evaluations and the first prospective experimental applications.^18^^,^^19^^,^^20^^,^^21^^,^^22^ Hence, these independent studies have provided a proof of principle for sequence-based compound design using transformer models,^18^^,^^19^^,^^20^^,^^21^^,^^22^ representing an exemplary prediction task that is difficult to address using standard (non-generative) machine learning methods. In these studies, sequence-to-compound mappings were learned to associate target sequences with specifically active compounds, reproduce known actives excluded from training, and/or generate novel actives.^18^^,^^19^^,^^20^^,^^21^ In addition, transformer models were conditioned on activity rules to predict potent compounds.^22^ Despite differences in system setups and calculation details, these investigations typically pre-trained transformers on large numbers of sequence-compound pairs, followed by fine-tuning on specific targets or target families (thus following the pre-training/fine-tuning protocol often applied in transformer modeling). However, explanations for the successful (re)generation of active compounds have remained elusive thus far.

In this work, we have used sequence-based compound design as a model system to elucidate the underlying learning characteristics of transformers. We have reasoned that sequence-based compound design based on learning sequence-to-compound mappings provides a suitable test system for careful control calculations evaluating well-defined compound data and sequence modifications to better understand how transformers arrive at their predictions, as reported in the following.

## Results

### Methodological framework

For our analysis, we pre-trained transformer models with the originally introduced architecture^7^ using sequence-compound pairs covering a large pharmaceutical target space based on alternative data partition schemes, as detailed in the methods section. Transformer variants were evaluated by determining their ability to reproduce known active compounds and core structures of known actives. The reproduction of core structures means that close structural analogs of known active compounds are generated. Following evaluation of the pre-trained models, fine-tuning was carried out on members of selected target protein families, including the CMGC Ser/Thr protein kinase family (CMGC) and their inhibitors and the G-protein-coupled receptor (GPCR) 1 family and their ligands. Notably, compounds with known activity against multiple targets, which are often available for protein families, might occur in training and test sets in different sequence-compound pairs (thus representing the only test compounds that are also found in training sets). We investigated in detail the influence of such multi-target (MT) compounds on the predictions. Then, by applying different types of sequence modifications, we generated a series of model variants for the analysis of sequence-dependent learning characteristics.

### Compound and core reproducibility

We first analyzed the reproducibility of test compounds and cores using transformer models pre-trained following sequence- or protein-family-based partitioning of sequence-compound pairs into training and test data. As a consequence of random partitioning of individual sequences, sequence-compound pairs of related proteins can be present in training and test data, thus representing similar sequences interacting with similar compounds. By contrast, family-based partitioning results in training and test sets comprising different families with no overlap and thus generally reduced sequence and compound similarity.

**Figure 1:**
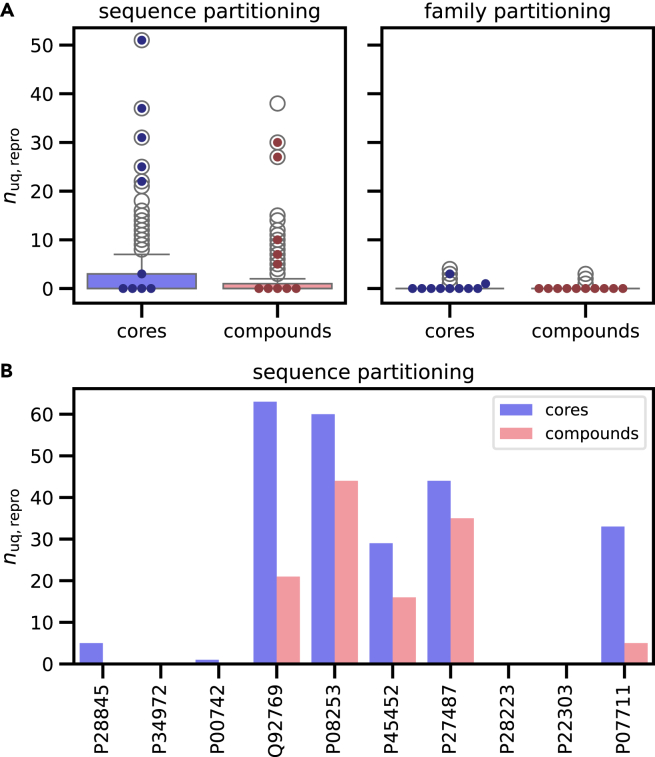
Exactly reproduced unique compounds and core structures (A) Boxplots (box: 1^st^ quartile, median, 3^rd^ quartile; whiskers: ±1.5× interquartile range) report the distribution of unique correctly reproduced (*n*_uq,repro_) test compounds and cores for 203 and 209 test sequences with more than 20 available compounds for pre-trained models based on sequence or family partitioning, respectively. For each test sequence, 2,500 output strings were sampled. Dots indicate the results for the 10 test sequences with the largest number of associated compounds. (B) For these 10 test sequences of the pre-trained model based on sequence partitioning, sampling was further extended to 5,000 output strings (as was the case for all test sequences discussed in the following), and the resulting numbers of unique exactly reproduced (*n*_uq,repro_) test compounds and cores are reported with UniProt accession numbers for the individual sequences.

Figure 1A reports the distribution of unique, exactly reproduced active test compounds and core structures for test sequences with more than 20 available compounds, leading to 203 and 209 test sequences for the models based on sequence- and family-based partitioning, respectively. The sequence-based pre-trained model generally reproduced test compounds and cores, with a mean of 2.0 and 2.8 compounds and cores per sequence, respectively. By contrast, the family-based model essentially failed to reproduce compounds and cores.

Figure 1B shows the results for the 10 test sequences with the largest number of available compounds following extended sampling using the sequence-based model, which exactly reproduced large numbers of up to ∼40 test compounds and ∼60 core structures for five and seven of the 10 test sequences, respectively. The results in Figure 1 demonstrate that compound/core reproducibility of the pre-trained models depended on the presence of closely related sequences and compounds in training and test data (that were reduced or eliminated by family-based partitioning).

We separately fine-tuned the sequence-based pre-trained model on members of the CMGC kinase and GPCR 1 families, respectively, and determined the compound/core reproducibility of the family-specific models. Figure S1 reports the number of unique, exactly reproduced cores for the pre-trained model and models separately fine-tuned for 10 members of the CMGC and GPCR 1 families with the largest number of associated compounds in the respective test sets. As anticipated, fine-tuning consistently increased the number of reproduced cores for sequences, for which compounds and cores were reproduced using the pre-trained model. Figure S1 also shows the most frequently reproduced core structures for members of the two target families.

Notably, control calculations revealed that compound reproducibility depended on transformer modeling, not merely on similarity among compounds sharing targets from the same family. The mean Tanimoto compound similarity values (calculated using binary Morgan fingerprints with a radius of 2 and a constant size of 2,048 bits) are 0.127 ± 0.048 (standard deviation) and 0.130 ± 0.050 for the GPCR and Ser/Thr kinase families, respectively. This reflects the limited similarity of compounds active against each of these target families. Furthermore, the mean compound similarity values per target-based dataset are 0.174 ± 0.111 and 0.153 ± 0.091 for the GPCR and Ser/Thr kinase families, respectively, and thus only marginally higher than the family-based average. Accordingly, reproducibility is not explainable based on large differences between intra- and inter-set similarity distributions. Overall, the low average similarity of active compounds does not preclude the presence of close structural analogs of individual active compounds in datasets, as further discussed below.

### Impact of MT compounds

**Figure 2:**
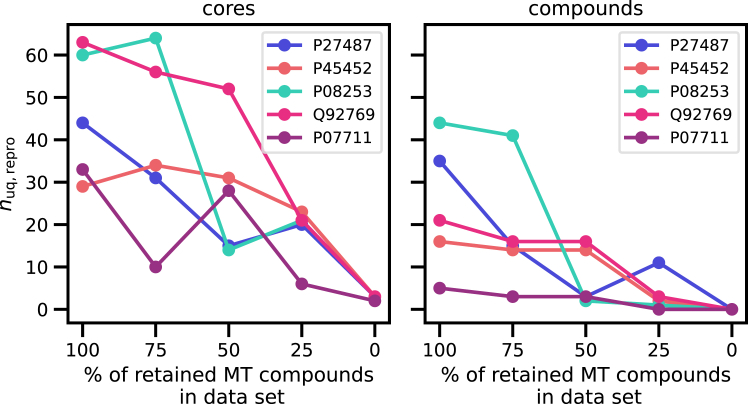
Influence of multi-target compounds Reported is the number of exactly reproduced unique (*n*_uq,repro_) cores (left) and compounds (right) for sequence-based models pre-trained in the presence of decreasing numbers of MT compounds. Results are shown for five test sequences with at least five exactly reproduced unique compounds for the model based on the original training set (100% MT compounds retained). For sequences, UniProt accession numbers are provided.

Sequence-compound pairs contain 29% of unique MT compounds, with a mean of 2.91 sequences per MT compound. We examined the influence of MT compounds on compound/core reproducibility. Therefore, we generated different versions of the sequence-based pre-trained model based on the stepwise cumulative removal of MT compounds from training data (that is, by converting MT compounds into single-target compounds; see methods). Figure 2 shows the results obtained for five test sequences, for which the original sequence-based pre-trained model exactly reproduced between five and 45 unique test compounds (and larger numbers of cores). While statistical fluctuations in reproduced numbers of compounds and cores were observed, for all five sequences, the number of reproduced compounds and cores decreased with decreasing amounts of MT compounds retained in the dataset. When all MT compounds were removed, no test compounds were exactly reproduced, and the number of reproduced cores declined to two to three per sequence.

As stated above, MT compounds were contained in training and/or test data in pairs with different target sequences. The lack of compound reproducibility in the absence of identical compounds in training and test data clearly indicates that the pre-trained model memorized compound structures encountered during training and regenerated them for test sequences that were similar to training sequences paired with these compounds. Analysis of the reproduced compounds further supported the importance of compound memorization. For the model based on the original dataset (100% of retained MT compounds), 96% of the reproduced compounds were already encountered during training because they were also associated with a different sequence. This ratio was reduced to 47% for the model based on the dataset with only 25% of retained MT compounds. However, these reproduced compounds have an average Tanimoto similarity of 0.84 to the nearest neighbor in the training set (calculated using binary Morgan fingerprints with a radius of 2 and a constant size of 2,048 bits). Thus, for most reproduced compounds, the exact compound or a highly similar one was available during training.

Taken together, these findings show that local compound and sequence similarity in training and test data (that is, similarity between individual instances) and compound memorization played a critically important role in reproducing compounds and core structures using the sequence-based pre-trained model, consistent with the lack of reproducibility of the family-based model discussed above.

### Sequence modifications

**Figure 3:**
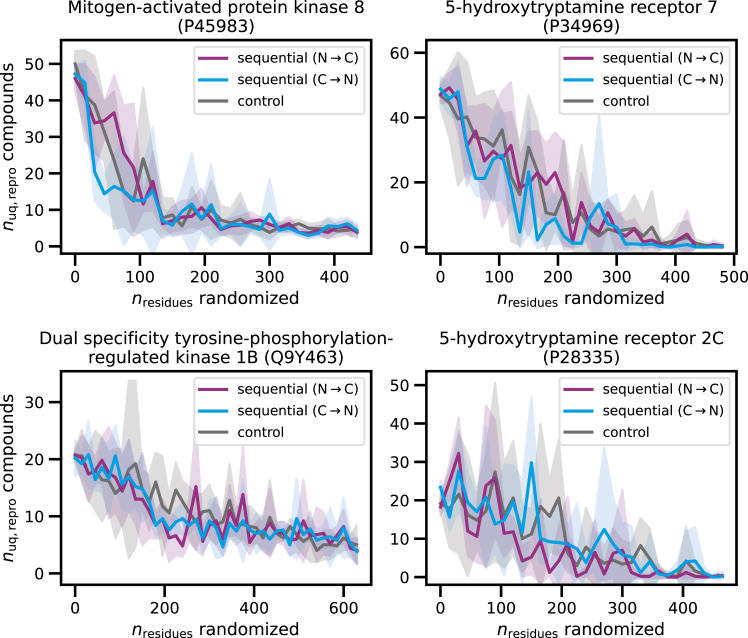
Exactly reproduced compounds for cumulatively randomized sequences The number of unique exactly reproduced compounds (*n*_uq,repro_ compounds) is reported for fine-tuned models following iterative cumulative randomization of 15-residue sequence segments (*n*_residues_ randomized) starting from the N to the C terminus, and vice versa. Control calculations iteratively randomized the same number of residues at randomly selected positions across the entire sequence. Results are shown for test sequences of two exemplary CMGC kinases and two GPCRs. The lines report the mean of five independent predictions and the shaded areas the standard deviation.

Next, we analyzed the effects of different sequence modifications on compound generation using fine-tuned CMGC and GPCR models. Cumulative sequence randomization of 15-residue segments in test sequences was applied from the N to the C terminus and in the opposite direction until the entire sequence was fully randomized (see methods). At each step, control calculations randomized the same number of residues at randomly selected positions across the entire sequence. Each sequence variant with an increasing number of randomized residues was used to predict test compounds. Figure 3 shows the compound reproducibility results for exemplary CMGC and GPCR sequences. For all sequences, we observed a decrease in the number of unique, exactly reproduced compounds for increasing numbers of randomized residues. The effects of randomization were essentially insensitive to the direction starting at the N or the C terminus and also closely comparable to the controls. For test sequence P45983 (top left), there was a sharp decline in the number of reproduced compounds for direction-/position-independent randomization of ∼150 residues to fewer than 10 reproduced compounds, followed by only small further reductions when the entire sequence was randomized.

A comparably steep reduction in the number of reproduced compounds was observed for P34969 (top right) when the first ∼200 residues were randomized, again followed by only small reductions when randomization was continued, also similar to P28335 (bottom right). We observed a more gradual direction-/position-independent decline in compound numbers across the entire sequence for Q9Y463 (bottom left) as a consequence of randomization. When the two GPCR sequences were fully randomized, no test compounds were reproduced. However, based on the fully randomized CMGC kinase sequences, up to five test compounds were still reproduced—an unexpected result. However, all of these reproduced compounds were found to be MT compounds, thus again indicating ligand memorization, as discussed above.

Taken together, the results for cumulative sequence randomization and positional controls clearly show that randomization of consecutive and non-consecutive residues had closely corresponding direction-/position-independent effects. As long as a sufficient proportion of the native sequence was retained, test compounds were reproduced. When ∼150–200 residues were randomized, up to ∼50% of the originally obtained test compounds continued to be reproduced, depending on the test sequence, regardless of the location of randomized residues in the sequence. Hence, the ratio of randomized versus original residues was the major determinant of compound reproducibility, but the identity of randomized or retained residues was largely irrelevant.

In light of these findings, we further investigated whether the fine-tuned models learned any sequence information characteristic of individual protein families or relevant for ligand binding. Therefore, known sequence motifs representing the conserved GPCR 1 and CMGC family signatures or the ATP binding site in kinases (which is targeted by most inhibitors) were masked by randomization or consistent replacement with alanine residues (see methods).

**Figure 4:**
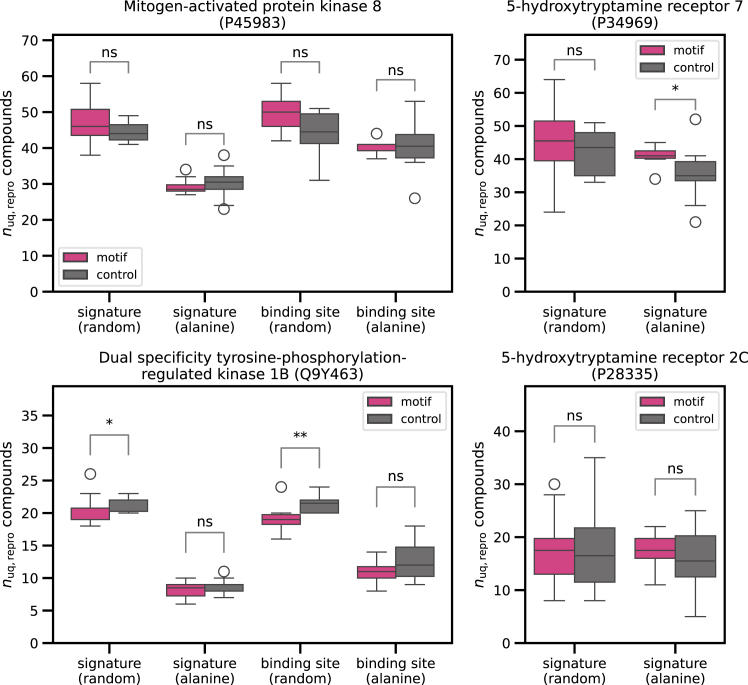
Exactly reproduced compounds for sequences with masked motifs The number of unique exactly reproduced compounds (*n*_uq,repro_ compounds) using fine-tuned models is reported after masking known sequence motifs (family signature or binding site motifs) through randomization of residues (random) or computational alanine scanning (alanine). Control calculations correspondingly replaced the same number of residues at random positions across the entire sequence. Results are shown for test sequences of two exemplary CMGC kinases and two GPCRs. The boxplots (box: 1^st^ quartile, median, 3^rd^ quartile; whiskers: ±1.5× interquartile range) report compound distributions across 10 independent prediction trials. For assessing statistical significance of differences in compound numbers between masking and control calculations, a two-sided Mann-Whitney U test was carried out to calculate *p* values: not significant (ns) *p* > 0.05, ∗*p* ≤ 0.05, ∗∗*p* ≤ 0.01, and ∗∗∗*p* ≤ 0.001.

Figure 4 reports the distribution of unique reproduced compounds for the fine-tuned models of the four sequences discussed above and differently modified sequence variants plus controls. For nine of the 12 test instances, no statistically significant differences between masked sequence motifs and random controls were observed. Since the masked sequence motifs were much smaller in size than the segments of randomized sequences leading to a significant reduction in reproduced compound numbers, as shown in Figure 3, the numbers of reproduced test compounds remained, in these cases, very close to the original ones observed for unmodified sequences.

The mostly observed absence of significant differences in compound reproducibility as a consequence of motif masking versus random sequence modifications indicates that model predictions did not depend on recognizing sequence motifs. We further examined the exceptions. For P34969, replacement of the family signature with alanine residues led to a statistically significant difference in the number of unique reproduced compounds (*p* = 0.0294 for 10 trials). However, the model reproduced more unique compounds when the motif was masked compared to random alanine replacements (which was likely due to statistical fluctuations between individual trials). In addition, for Q9Y463, randomization of the family signature and the ATP site motif resulted in significantly fewer reproduced compounds compared to the random controls (with *p* = 0.0498 and 0.0055 for 10 trials each, respectively). This was the only observation indicating motif relevance, but it was not reproducible by alternative motif masking through alanine replacements (again likely reflecting an influence of statistical fluctuations). Taken together, these findings indicate that the fine-tuned models did not learn specific (biologically relevant) sequence motifs for compound generation, consistent with the results of cumulative sequence randomization.

## Discussion

Transformers have become preferred architectures for language models and have been adopted in many areas, including the molecular sciences. In drug discovery and design, they are used for applications such as generative modeling of diverse chemical structures or the prediction of molecular properties of new active compounds. The versatility of transformers in learning different types of molecular representations and mappings enables off-the-beaten-path applications that are difficult or impossible to address with standard machine learning or structure generation methods. Among these is protein-sequence-based design of active compounds, which has recently been investigated in several studies. However, rationalizing predictions of transformer models is a difficult task, and the development of XAI approaches for transformer networks is still in its early stages.^11^ Notably, the black-box character of deep neural network architectures, such as transformers, limits their impact on experimental design in interdisciplinary research, which represents a topical issue in machine learning.

In this work, we have used sequence-based compound design as a model system to explore and better understand the learning characteristics of molecular transformer models. We have reasoned that sequence-based compound design enabled detailed control calculations for transformer predictions based on specific compound data or sequence modifications, as reported herein. Therefore, we have trained transformers on sequence-compound (input-output) pairs, compared alternative pre-trained models based on different data-partitioning schemes, and fine-tuned models on sequence-compound pairs of proteins belonging to two major pharmaceutical target families in the presence and absence of defined modifications.

Our analysis reveals that the ability of transformer models to reproduce active test compounds or core structures depended on several factors. An essential condition of compound/core reproducibility was detectable similarity of sequence-compound pairs in training and test data, as revealed by the failure of models pre-trained following family-based sequence partitioning or conversion of MT compounds with multiple sequence-compound pairs into single-target compounds. The latter data modification showed that no test compounds were exactly reproduced if they were not encountered during training in different compound-sequence pairs, hence revealing compound memorization effects. Fine-tuned models for individual kinases even reproduced a limited number of compounds based on random sequence input if multiple copies of these compounds were present in training data pairs (causing a form of model over-fitting). Furthermore, the results of cumulative sequence randomization and control calculations clearly indicate that entire sequences were not required for successful compound/core predictions. Instead, models were tolerant to sequence randomization as long as a sufficient proportion of the original sequence remained (∼60%). However, the composition and location of these sequence subsets were not important. Moreover, masking of family signature or binding site sequence motifs did not compromise compound reproducibility.

Taken together, these findings demonstrate that transformer-based sequence-to-compound modeling was, in the presence of essential sequence and molecular similarity, purely statistically driven and that the models did not learn sequence motifs characteristic of protein families or relevant for ligand binding. Moreover, compound memorization effects played a major role for reproducibility.

Predictions purely depending on statistical associations are valid from a machine learning perspective. However, given their statistical nature, care should be taken not to over-interpret them. This is not without precedent. For instance, predictions of compound potency based on protein-ligand interaction diagrams using graph neural networks were previously shown to be mostly determined by ligand memorization effects^23^^,^^24^—and not by the putative ability of these networks to learn protein-ligand interactions, as frequently claimed in the literature.^23^ Such over-interpretations often lead to Clever Hans predictors,^25^ that is, models arriving at desirable results for reasons other than those anticipated or claimed. Based on such predictions, incorrect causal relationships are likely to be proposed in machine learning.^24^^,^^25^ For transformer CLMs, this can be avoided, at least for the applications reported herein, by considering the purely statistical nature of the predictions, independent of potential biological and/or chemical foundations. Accordingly, transformer models might be applicable to prediction tasks in the absence of an underlying sound scientific rationale as long as statistical correlations can be detected, for which sequence-based compound design provides an instructive example. Of course, based on our initial investigation, we cannot generalize the observed transformer learning characteristics for molecular design. Depending on the application, other learning characteristics might be observed. Hence, additional studies exploring transformer learning in chemistry will be required before more general conclusions can be drawn.

## Methods

### Compounds, sequences, and computational representations

We extracted active compounds with a molecular weight of max. 1,000 Da from ChEMBL (v.34).^26^ Only numerically specified activity measurements resulting from a direct protein binding/inhibition assay at the highest level of confidence (ChEMBL confidence score 9) were considered. Compounds were discarded if multiple measurements for the same target were available that did not fall into the same order of magnitude. In addition, compounds with activity annotations for anti-targets, assay interference potential, or aggregator likelihood were removed using public filters.^27^^,^^28^^,^^29^ For each remaining compound, all available target annotations were recorded, thereby distinguishing between compounds with single-target or MT activity. Accordingly, in contrast to single-target compounds, MT compounds were paired with two or more sequences. Target sequences with a maximum length of 1,000 amino acids were obtained from the UniProt database.^30^

The resulting dataset consisted of 100,626 unique compounds, the sequences of 1,419 unique targets these compounds were active against, and 156,762 sequence-compound pairs (interactions), with a mean of 110 compounds per sequence and mean activity annotations of 1.56 targets (sequences) per compound.

For compounds, we generated canonical nonisomeric SMILES strings, which were tokenized using a regular expression developed by Schwaller et al.,^31^ followed by one-hot-encoding. Additionally, [start], [end], and [pad] tokens were used to indicate the start and end of a compound or sequence and generate uniform sequence encodings of the maximum length, respectively. Positional encoding was applied, as introduced for the original transformer architecture.^7^ Sequences were represented using the IUPAC amino acids code, followed by one-hot encoding of individual residues.

### Data partitioning

We applied two different partitioning schemes to generate training/test sets with a 70%/30% data ratio. In sequence-based partitioning, individual sequences and associated compounds were randomly divided. In family-based partitioning, sequences and associated compounds were divided based on protein families according to the UniProt classification scheme.^30^ Both partitioning schemes led to non-overlapping training and test sets.

### MT compounds

Additional datasets were prepared to analyze how the presence or absence of MT compounds might influence model performance. Therefore, we iteratively converted MT compounds available in the entire datasets into single-target compounds by removal of all but one corresponding sequence-compound pair, resulting in a stepwise decrease of MT dataset compounds. For instance, for an MT compound with activity against three targets, two of the sequence-compound pairs were randomly removed such that only one of the pairs remained. This procedure was carried out following sequence-based partitioning and ensured that for an MT compound, only a single sequence-compound pair remained either in a training or test set.

### Sequence modification

We modified input sequences in different ways to identify residues or sequence segments affecting the predictions. These modifications included randomization of residues (that is, a given amino acid was randomly replaced with likelihoods derived from the relative frequency of occurrence of amino acids in the entire dataset) and computational alanine scanning (that is, amino acids were consistently replaced with alanine).

### Cumulative randomization

Cumulative randomization was carried out with a segment size of 15 residues, that is, starting from the N or the C terminus of a given sequence, the first (last) 15 residues were randomized, followed by the next 15 residues, thus randomizing 30 subsequent residues. We then continued cumulative randomization of 15-residue segments until the entire sequence was randomized (from the N or the C terminus in opposite directions). As a control calculation, at each iteration, the same number of amino acids was randomized at positions randomly selected across the entire sequence. Given the statistical nature of the control, each randomization calculation was repeated five times.

### Masking of sequence motifs

To test the potential influence of specific sequence motifs (such as residues forming a binding site or a sequence signature of a protein family) on the predictions, selected motifs were either randomized or replaced with alanine residues. As a control, the same number of amino acids was randomized or replaced with alanine at positions randomly selected across the entire sequence. To account for statistical fluctuations associated with the modification of sequence motifs of limited size, each control calculation was repeated 10 times.

### Sequence motifs

We obtained the following sequence motifs from ProSite^32^^,^^33^ for masking. For 5-hydroxytryptamine receptor 7 and 5-hydroxytryptamine receptor 2C, belonging to the GPCR 1 family, the family signature motif was selected (residues 168–184 and 140–156, respectively). In addition, for mitogen-activated protein kinase 8 and dual-specificity tyrosine-phosphorylation-regulated kinase 1B, the sequence motif of the ATP binding site (residues [32–40, 55] and [117–125, 140], respectively) and the Ser/Thr protein kinase active site signature (residues 147–159 and 235–247, respectively) were selected.

### Model derivation, fine-tuning, and evaluation

We implemented the originally reported encoder-decoder transformer architecture^7^ using PyTorch^34^ and PyTorch Lightning^35^ with the hyperparameters listed in Table S1. Pre-training and fine-tuning of the model employed a cyclic learning rate scheduler using the hyperparameters reported in Table S2. Training was carried out with sequence-compound pairs (with sequence and compound data processed by the encoder and the decoder, respectively). We employed the cross-entropy loss as the loss function. For testing, the sequences were used as input for the encoder, and the decoder was initialized with the start token to recursively generate the following tokens. Multinomial sampling was employed with a temperature setting of 1. For the test sequences with more than 20 available compounds, 2,500 output strings were sampled. To further analyze the test sequences discussed in the text, sampling was consistently extended to 5,000 strings.

Following transformer pre-training, fine-tuning was carried out through additional training using only compound-sequence pairs from a specific protein family extracted from the corresponding training set. Fine-tuning yielded multiple family-specific models.

We evaluated the different model variants by determining the number of unique exactly reproduced test compounds available for a given test sequence (target) and the number of reproduced core structures (scaffolds) contained in test compounds. Cores were extracted from compounds using the compound-core relationship (CCR) algorithms.^36^ The CCR determines the invariant core structure for multiple compounds, forming a unique analog series.

### Data analysis and visualization

We analyzed all data with public Python packages, including NumPy,^37^ SciPy,^38^ and Pandas.^39^ Visualizations were generated using matplotlib^40^ and seaborn.^41^ Compounds and their representations were processed with RDKit,^42^ which was also used to display compound structures.

## Resource availability

### Lead contact

Requests for further information and resources should be directed to and will be fulfilled by the lead contact, Jürgen Bajorath (bajorath@bit.uni-bonn.de).

### Materials availability

This study did not generate new unique reagents.

### Data and code availability

Table 1 shows the data and software resources used in this study. Datasets, model checkpoints, training scripts, and analysis scripts are available at https://doi.org/10.5281/zenodo.16322841.^43^

**Table 1:** Key resources table

Reagent or resource	Source	Identifier
**Deposited data**		

Model checkpoints, training and inference scripts, analysis scripts, raw and analyzed data	this work	https://doi.org/10.5281/zenodo.16322841

**Software and algorithms**		

NumPy	Harris et al.^37^	https://doi.org/10.1038/s41586-020-2649-2
SciPy	Virtanen et al.^38^	https://doi.org/10.1038/s41592-019-0686-2
Pandas	Pandas Development Team^39^	https://doi.org/10.5281/zenodo.13819579
PyTorch	Ansel et al.^34^	https://doi.org/10.1145/3620665.3640366
PyTorch Lightning	Falcon et al.^35^	https://doi.org/10.5281/zenodo.3828935
Matplotlib	Hunter^40^	https://doi.org/10.1109/MCSE.2007.55
Seaborn	Waskom^41^	https://doi.org/10.21105/joss.03021
RDKit	Landrum et al.^42^	https://doi.org/10.5281/zenodo.13469390

**Other**		

ChEMBL v.34	Zdrazil et al.^26^	https://doi.org/10.1093/nar/gkad1004
PROSITE	Sigrist et al.^32^ and Castro et al.^33^	https://doi.org/10.1093/nar/gks1067 and https://doi.org/10.1093/nar/gkl124

Data and software resources used in this study.

## Acknowledgments

We thank Martin Vogt for providing code for the compound-core relationship algorithm. We also thank Martin Vogt, Tiago Janela, and Alec Lamens for helpful discussions. J.P.R. thanks the German Academic Scholarship Foundation (Studienstiftung des deutschen Volkes) for financial support.

## Author contributions

Conceptualization, J.B.; investigation and formal analysis, J.B. and J.P.R.; data curation, software, and visualization, J.P.R.; writing – original draft and writing – review & editing, J.B. and J.P.R.

## Declaration of interests

The authors declare no competing interests.

## Supplemental information

Document S1. Figure S1 and Tables S1 and S2

Document S2. Article plus supplemental information
