# Supplementary material for: Unraveling learning characteristics of transformer models for molecular design
Source: Patterns (N Y). 2025 Oct 14;6(12):101392. doi: 10.1016/j.patter.2025.101392 (PMC12745979; doi:10.1016/j.patter.2025.101392)
Supplement: Document S1. Figure S1 and Tables S1 and S2 [file mmc1.pdf]

Patterns, Volume *6*

Supplemental information

Unraveling learning characteristics of transformer models for molecular design

Jannik P. Roth and Jürgen Bajorath

<u>Table-of-Contents</u>

Supplementary Figure S1

Supplementary Table S1

Supplementary Table S2

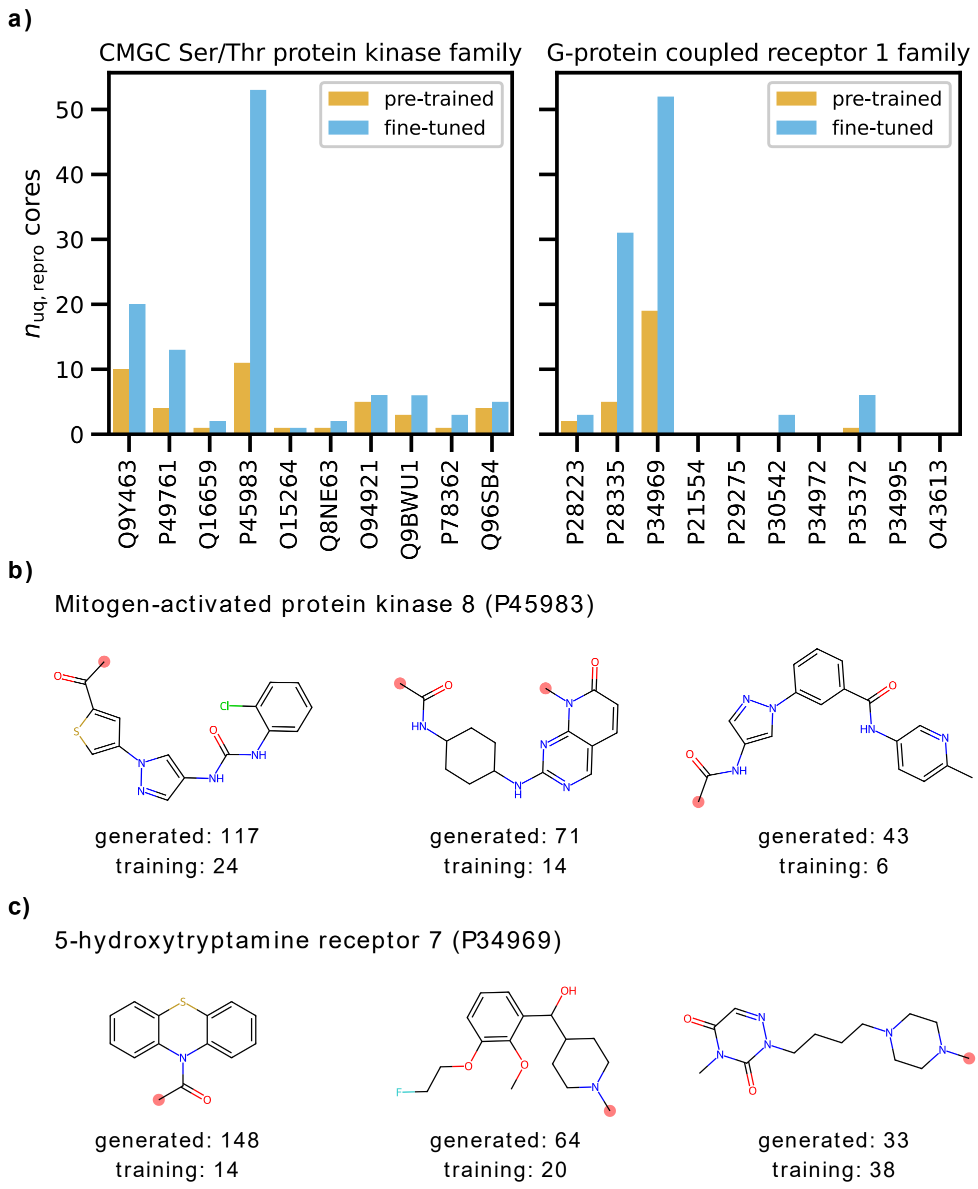

Figure S1. Exactly reproduced core structures before and after fine-tuning. **a**) reports the number of unique exactly reproduced test cores ($n_{uq,repro}$ cores) by the pre-trained and fine-tuned model for the 10 test sequences with largest numbers of associated compounds from the CMGC Ser/Thr protein kinase family (left) and GPCR 1 family (right). For sequences, UniProt accession numbers are provided. **b**) and **c**) show the three most frequently reproduced cores by the fine-tuned models for an exemplary kinase and GPCR, respectively. For each core, the number of fine-tuning training compounds having the core (training) and the number of generated compounds (generated) are reported. In cores, substitution sites are indicated with red circles.

Table S1. Transformer hyperparameters.

**Parameter**	**Value**
N (encoder layers)	6
N (decoder layers)	6
N (attention heads)	4
Model dimension	256
Feedforward dimension	512

Hyperparameters of the transformer model were implemented using PyTorch's *nn.transformer* class.

Table S2. Hyperparameters for the learning rate scheduler.

**Parameter**	**Pre-training**	**Fine-tuning**
base learning rate	1.5e-5	1.5e-5
max learning rate	1.5e-4	1.0e-4
step size up	30	15
step size down	170	85
max epochs	600	100
mode	exp range	exp range
gamma	0.9965	0.9965

Hyperparameters of the learning rate scheduler were implemented using PyTorch's PyTorch's *optim.lr_scheduler.CyclicLR* class. For pre-training and fine-tuning, the batch size was set to 1 due to memory requirements but the gradient was accumulated over a batch size of 64.
